# Supplementary material for: Learning collective multicellular dynamics with an interacting mean field neural SDE model
Source: PLoS Comput Biol. 2026 Jan 21;22(1):e1013916. doi: 10.1371/journal.pcbi.1013916 (PMC12854464; doi:10.1371/journal.pcbi.1013916)
Supplement: S1 Text — (DOC) [file pcbi.1013916.s001.doc]

S1 Text. Computational Cost

We assessed the scalability of scIMF on large-scale data by measuring both total runtime and peak memory usage.

Specifically, we subsampled the MEF dataset (236,285 cells) to construct four benchmark datasets containing 10%, 20%, 50%, and 100% of the cells. Under a fixed mini-batch size and a fixed number of training epochs, scIMF scaled well up to the full dataset with over 230K cells (S2 Fig). Overall, the mini-batch strategy within the deep learning framework substantially reduced both the runtime and memory footprint of scIMF.

We further compared the computational time and memory consumption of scIMF with other methods on the ZB dataset (S1 Table). These results show that scIMF achieves competitive efficiency relative to other neural ODE/SDE-based models.

Note that all benchmarks were run on a single GPU (NVIDIA GeForce RTX 4090, 24 GB VRAM), and all methods were evaluated using their default settings.
