# Supplementary material for: Learning collective multicellular dynamics with an interacting mean field neural SDE model
Source: PLoS Comput Biol. 2026 Jan 21;22(1):e1013916. doi: 10.1371/journal.pcbi.1013916 (PMC12854464; doi:10.1371/journal.pcbi.1013916)
Supplement: S2 Text — (DOC) [file pcbi.1013916.s002.doc]

S2 Text:Robustness of scIMF to the of attention heads (*H*) and the PCA dimension (*r*)

We evaluated the robustness of scIMF with respect to two key hyperparameters: (i) the number of attention heads (*H*), and (ii) the PCA embedding dimension (*r*), on the prediction accuracy of the “Hard task” on the ZB dataset. Prediction error was quantified as the discrepancy between the predicted and true gene expression profiles using *ℓ*₁- and *ℓ*₂-Wasserstein distances (*W*₁ and *W*₂).

We first varied the number of attention heads from *H* = 2 to *H* = 10 while keeping all other settings fixed. As shown in S2 Table, the performance of scIMF remains stable across different choices of *H*.

We then assessed robustness to the PCA dimension by varying the number of top principal components, *r* ∈ {20, 30, 40}, on the ZB dataset and comparing five methods — scIMF, scNODE, PRESCIENT, PI-SDE, and MIO-FLOW — each run with its default settings for every selected *r*. Note that when the feature-space dimension changes (e.g., *r*=20 vs. *r*=30 or *r*=40), the Wasserstein distances $W_{p}({\mathbb{\mathbb{R}}}^{r})$ are not directly comparable across different *r*; higher-dimensional embeddings tend to yield larger *W*₁/*W*₂ values simply because transport occurs in a higher-dimensional space. We therefore perform quantitative cross-method comparisons only within a fixed PCA dimension. As shown in S3 Fig, scIMF consistently attains low *W*₁/*W*₂ scores relative to all baselines across the three settings (*r*=20, *r*=30, and *r*=40). These results indicate that our conclusions about the relative performance of scIMF are robust to the choice of PCA dimension.
