# Supplementary figures and images for: Learning collective multicellular dynamics with an interacting mean field neural SDE model
Source: PLoS Comput Biol. 2026 Jan 21;22(1):e1013916. doi: 10.1371/journal.pcbi.1013916 (PMC12854464; doi:10.1371/journal.pcbi.1013916)

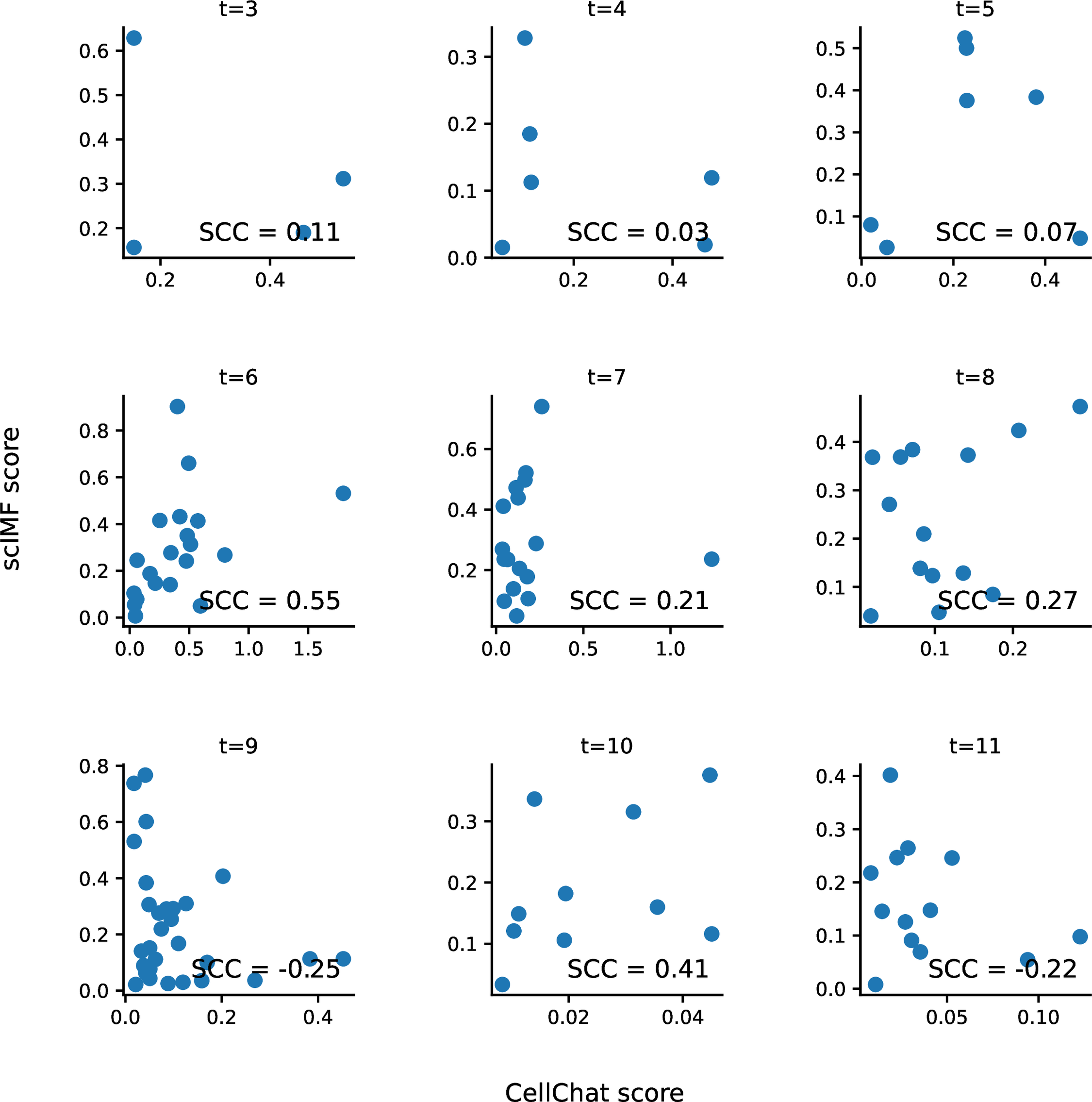

Supplement: S1 Fig — For each developmental time point t=3–11, scatter plots compare the CellChat ligand–receptor–based interaction weights (x-axis) with the scIMF attention scores (y-axis). Only directed interactions between distinct cell-type pairs that pass the applied filters (CellChat score ≥0.005; scIMF attention score ≥0.001) are shown. Each panel corresponds to a single time point and reports the Spearman correlation coefficient (SCC) quantifying the agreement between the two methods. Note that earlier time points (t≤2) are excluded due to insufficient numbers of cell types and non-trivial interactions for reliable comparison. (TIFF) [file pcbi.1013916.s005.tif]

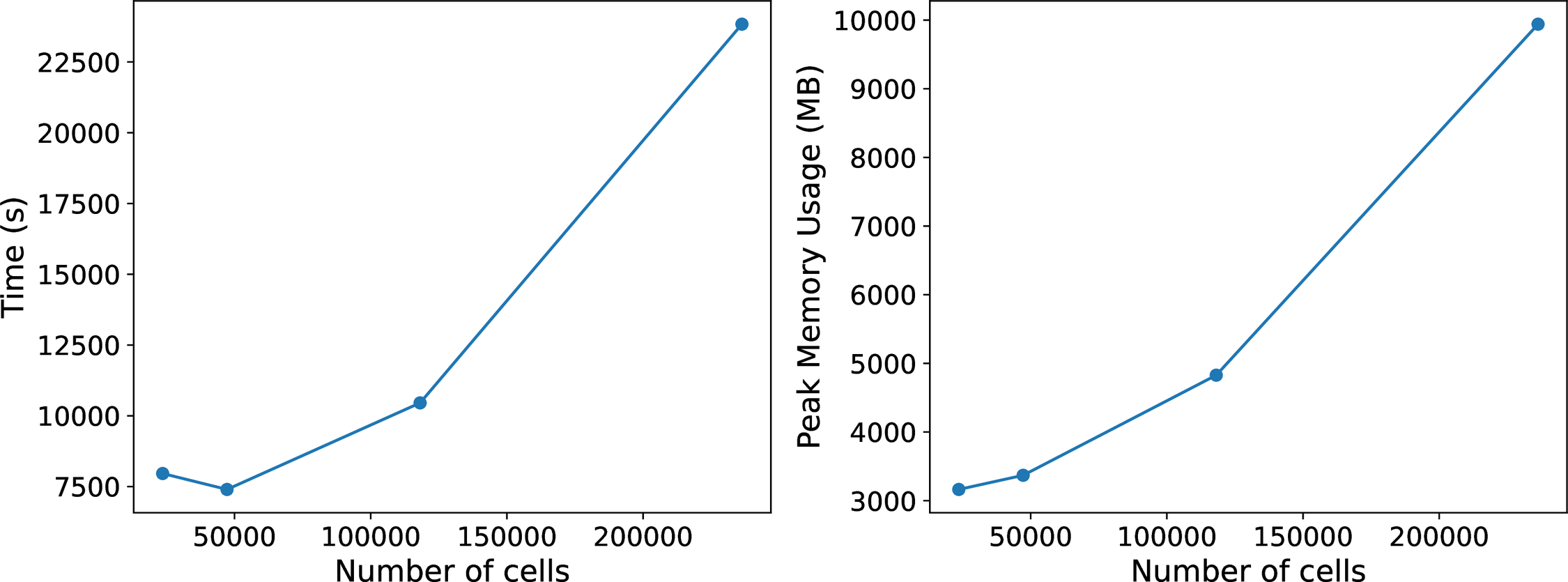

Supplement: S2 Fig — Total runtime (left) and peak memory usage (right) of scIMF for different data sizes obtained by subsampling 10%, 20%, 50%, and 100% of the 236,285 cells in the MEF dataset. (TIFF) [file pcbi.1013916.s006.tif]

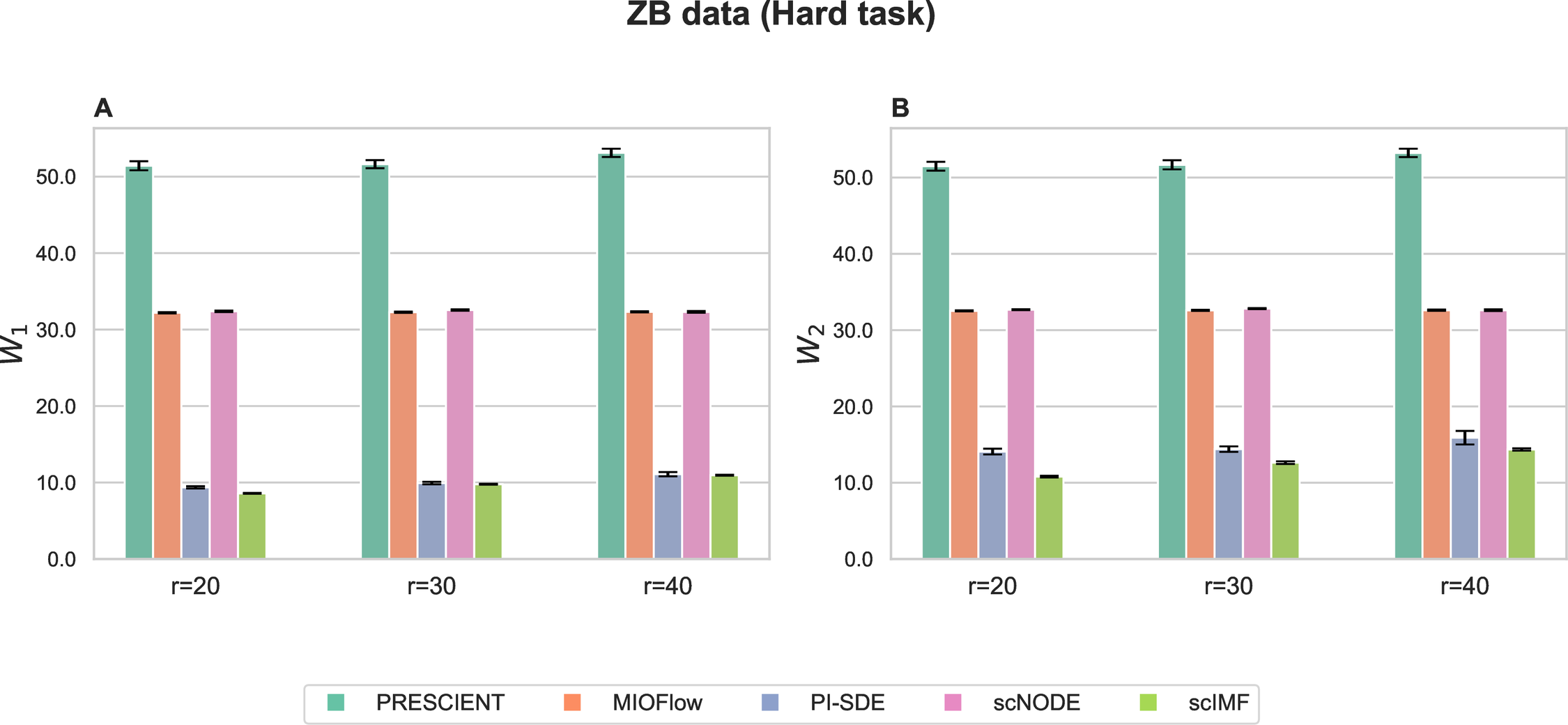

Supplement: S3 Fig — Evaluated W1 and W2 scores of scIMF and four comparison methods for different choices of PCA dimension r on the hard task of the ZB dataset; mean ± standard deviation is reported for all metrics. (TIFF) [file pcbi.1013916.s007.tif]
