# Supplementary material for: Learning collective multicellular dynamics with an interacting mean field neural SDE model
Source: PLoS Comput Biol. 2026 Jan 21;22(1):e1013916. doi: 10.1371/journal.pcbi.1013916 (PMC12854464; doi:10.1371/journal.pcbi.1013916)
Supplement: Attachment — Submitted filename: Review Report for PCOMPBIOL-D-25-01919.pdf [file pcbi.1013916.s008.pdf]

Review Report for PCOMPBIOL-D-25-01919

This paper presents scIMF, a deep generative model for learning collective multicellular dynamics from temporal single-cell RNA-seq data. The method is conceptually grounded in the McKean–Vlasov stochastic differential equation framework, which provides a principled way to model population-level interactions. The incorporation of a cell-wise attention mechanism to capture nonlocal and asymmetric cell-cell interactions is an appealing idea and addresses a limitation of many existing approaches.

The work is technically solid and demonstrates promising performance on several temporal scRNA-seq datasets, particularly in reconstructing gene expression at unobserved time points and inferring cellular velocities. The results suggest that scIMF can capture meaningful biological dependencies and provide interpretable insights into multicellular systems.

Overall, this is a well-motivated and technically interesting study that contributes a novel perspective to modeling cell-cell interactions in dynamic single-cell data. The approach is promising but would benefit from more experimental justification and practical discussion of its computational and biological limitations. Here are the detailed comments.

1. The authors emphasize temporal single-cell RNA sequencing, but it is unclear what exactly "temporal" refers to in this context. Currently, there are two main types of scRNA-seq data: (1) data collected from cells at a single time point from one individual, which require trajectory or pseudotime inference to reconstruct temporal information; and (2) data collected at multiple time points from different experimental individuals. If the authors refer to the first type, is scIMF sensitive to errors or fluctuations in pseudotime inference? If the

second type is used, is scIMF sensitive to inter-individual variation among different experimental subjects? If another type of data is used, the authors should briefly clarify this in the manuscript.

2. Since cellular dynamics evolve continuously over time while the observed data are discrete, are there potential latent states that are unobserved or unmodeled? Would this omission introduce bias when computing optimal transport distances?
3. The algorithm employs modeling frameworks such as the McKean–Vlasov stochastic differential equation and mean-field games. Although the empirical results support their effectiveness, it would be helpful if the authors could provide biological intuition to explain why these methods yield better results in this context.
4. The authors mention dimensionality reduction in preprocessing, but the choice of method and dimensionality is not further discussed. How sensitive are the results to these choices? How should one select the dimensionality in practice?
5. Some symbols in the equations are not clearly defined or explained—for instance, $\rho(x,t)$. Also, in Equation (2), $x_{t_0} = \rho_{t_0}$, the left-hand side is a numerical value, while the right-hand side is a distribution. Even if it represents a degenerate (single-point) distribution, the notation seems nonstandard and should be clarified.
6. From Fig.2, the results on the first two *in vivo* datasets appear better than those on the *in vitro* dataset. Is there any biological or methodological explanation for this difference?
7. The brief description of the empirical datasets is placed on page 11. It might be better to move this section earlier, as some parts of the manuscript (for example, the discussion of “held-out time points” on page 4) are difficult to follow without this context.
