# Supplementary material for: Learning collective multicellular dynamics with an interacting mean field neural SDE model
Source: PLoS Comput Biol. 2026 Jan 21;22(1):e1013916. doi: 10.1371/journal.pcbi.1013916 (PMC12854464; doi:10.1371/journal.pcbi.1013916)
Supplement: Attachment — Submitted filename: Response_to_Reviewers.pdf [file pcbi.1013916.s009.pdf]

Responses to Reviewers:

We would like to thank the reviewers for thoughtful comments and valuable suggestions on our manuscript. The manuscript has been fully revised by following the reviewers' comments and suggestions, making our results more solid and convincing.

The point-by-point responses to the reviewers' comments and suggestions are as follows and our responses are in red color.

Reviewer #1:

Overall comments

R1: This manuscript presents scIMF, a novel deep generative model for learning collective multicellular dynamics from temporal single-cell RNA-seq data. The work is highly timely and addresses a critical gap in the field: the integration of cell-cell interactions (CCIs) into dynamic models of cellular behavior. The authors combine the McKean-Vlasov stochastic differential equation framework with a Transformer-based attention mechanism to capture non-local and asymmetric interactions at single-cell resolution.

Response: We thank the reviewer for their positive assessment of scIMF and for highlighting the novelty of applying the McKean-Vlasov framework to single-cell data.

Major comments

R1.1: Please add analysis to the time complexity of the proposed algorithm. Is this method applicable to large-scale single-cell RNA-seq datasets? It would be helpful to show the running time.

Response:

We sincerely thank the reviewer for raising this crucial point regarding the computational complexity and scalability of our method. We agree that demonstrating the applicability of scIMF to large-scale datasets is essential.

In the revised manuscript, we have added a detailed theoretical complexity analysis in the **Methods** section. We clarify that while standard self-attention

is quadratic, our mini-batch implementation effectively linearizes the cost with respect to the total number of cells N. The revised text reads:

> "Since self-attention is $\mathcal{O}(N^2)$ in the number of cells N, scIMF adopts a mini-batch strategy to scale to large datasets by sampling B cells per iteration ($B \ll N$ in practice). In each iteration, the computational time complexity is dominated by two components: (i) the transformer-based drift term, which is $\mathcal{O}(B^2)$, and (ii) the computation of the Sinkhorn optimal transport loss between the predicted and observed cell distributions, which is $\mathcal{O}(BN)$. Consequently, for fixed batch size B (and a fixed number of Sinkhorn iterations), the overall complexity of scIMF grows approximately linearly with N, since attention is confined to fixed-size batches and the only N-dependent term arises from the entropically-regularized OT matching. The memory consumption likewise scales linearly with N, as evidenced by the approximately linear increase in time and memory usage with the total number of cells used in MEF dataset (Supplementary Text 1, Table S1 and Fig. S2)." (Lines 447–458)

To empirically validate this analysis, we measured the running time and memory usage on the MEF dataset. These results are now detailed in **Supplementary Text 1**, **Table S1**, and **Figure S2**.

Our empirical results confirm that the running time and memory consumption increase linearly with the number of cells N. This demonstrates that scIMF scales efficiently and is well-suited for analyzing large-scale single-cell RNA-seq datasets.

R1.2: The model infers interactions directly from the data without prior biological knowledge. Could the framework be extended to incorporate known biological priors (e.g., ligand receptor interactions or specific pathway information), and if so, how might that be implemented to potentially improve validity of the algorithm?

Response:

We thank the reviewer for this insightful suggestion. We agree that while prior-free learning is a strength of scIMF, integrating known biological interactions could further enhance the model's validity and interpretability.

In the revised manuscript, we have expanded the **Discussion** section (Page

12) to explicitly outline how the framework can be extended to incorporate biological priors (such as ligand–receptor pairs). The revised text is as follows:

> "Although the primary scIMF model is trained in a prior-free manner, its architecture naturally accommodates biological priors within the intercellular drift term. Conceptually, the interaction dynamics can be decomposed as:
>
> $$\mathbf{f}_{\text{inter}} = (1 - \alpha)\mathbf{f}_{\text{global}} + \alpha\mathbf{f}_{\text{LR}}.$$
>
> Here, the global component $\mathbf{f}_{\text{global}}$ corresponds to our current implementation, in which intercellular influences are inferred purely from data by aggregating gene expression across the population. The additional ligand–receptor term, $\mathbf{f}_{\text{LR}}$, provides a principled mechanism for incorporating curated biological knowledge. Concretely, given cell-by-ligand and cell-by-receptor matrices, $\mathbf{f}_{\text{LR}}$ can be implemented as a cross-attention module mapping ligand profiles of sender cells to receptor profiles of receiver cells. Curated interaction databases are then used to mask or reweight the attention probabilities, ensuring that only biologically plausible sender–receiver pairs contribute to the overall drift. The contribution of this prior-guided branch is modulated by a mixing coefficient α; when this coefficient is set to zero, the model recovers the exact formulation of the original, prior-free scIMF." (Lines 300–313)

R1.3: Line 157, "For example, blastomere subpopulations exhibit diverse interaction patterns, suggesting complex early-stage dynamics. By late stages (t = 7, 8, 9, 10), intra-cell-type interactions become more homogeneous, particularly in neural cells, reflecting their transition to functional specialization." What's meaning of this sentence? Is there cell type information in Fig. 5A? Please elaborate the analysis and explain the figure clearly.

Response:

We thank the reviewer for requesting clarification regarding the interpretation of Fig. 5A and the description of cell-type dynamics. We apologize if the original figure description was not sufficiently clear regarding the visualization of cell types.

(1) Clarification of Cell Type Information in Fig. 5A. We wish to clarify that cell type information is indeed present in Fig. 5A. The cells along both the

rows (target/query) and columns (source/key) of the heatmaps are **grouped and ordered by their annotated cell types**. This grouping is visually indicated by the **colored side bars** along the axes, which correspond to the cell-type legend provided in Fig. 5B.

(2) Interpretation of Heterogeneity vs. Homogeneity.

Regarding the sentence in question, our analysis refers to the visual patterns within these cell-type blocks:

- **Early Stages (e.g., Blastomeres):** The attention scores within the blastomere block exhibit high variance. This indicates **heterogeneity**, suggesting that even within the same cell type annotation, individual cells are interacting differently, reflecting early developmental plasticity and complex dynamics.
- **Late Stages (e.g., Neural cells):** The attention scores within the neural cell blocks become visually smooth and uniform. This indicates **homogeneity**, suggesting that as cells differentiate and commit to a specific lineage, their communication patterns become synchronized and stable, reflecting functional specialization.

To make this clear to readers, we have revised the captions for Fig. 5 (as well for Fig. 6), and we have elaborated on the analysis in the main text.

Revised Fig. 5 Caption (Page 9):

> "(A) Heatmaps of cell-wise attention score matrices across developmental time points in the ZB dataset. For each matrix, rows correspond to target cells (queries) and columns to source cells (keys); colors denote the row-normalized attention strength from each source cell to each target cell. Cells along both axes are grouped and ordered by annotated cell type, with colors indicated by the side bars."

Revised Main Text (Page 8):

> "In early development ($t = 0, 1, 2, 3$), intra-cell-type attention scores often show high heterogeneity, characterized by high variance in attention weights within specific cell-type blocks. For example, blastomere subpopulations exhibit diverse interaction patterns, suggesting complex early-stage dynamics where cells of the same annotation may possess varying communicative potentials. By late stages ($t = 7, 8, 9, 10$), intra-cell-type interactions become more homogeneous, appearing as visually uniform blocks, particularly in neural

> cells; this reflects their transition to functional specialization and synchronized collective behavior.” (Lines 182–190)

We believe these revisions explicitly link the visual patterns in the heatmap to the underlying biological interpretation.

Reviewer #2:

Overall comments

R2: The manuscript by Jiang et al. presents scIMF, a novel deep generative model for learning the dynamics of cell populations from time-series single-cell RNA sequencing (scRNA-seq) data. The central contribution of this work is the explicit modeling of cell-cell interactions (CCIs), a factor often ignored by existing methods that treat cells as independent agents. The authors achieve this by framing multicellular dynamics within the McKean-Vlasov Stochastic Differential Equation (MV-SDE) framework, where each cell's trajectory is influenced by the mean field (empirical distribution) of the entire population. To capture specific, non-local, and potentially non-reciprocal interactions, the model innovatively employs a cell-wise attention mechanism. Through comprehensive benchmarking on three distinct datasets, the authors demonstrate that scIMF outperforms current state-of-the-art methods in predicting gene expression at unobserved time points and in inferring cellular velocities. Furthermore, the model's learned attention scores provide biologically interpretable insights, distinguishing between asymmetric, non-equilibrium dynamics in vivo and symmetric, quasi-equilibrium dynamics in vitro.

General Assessment This is a well-written and technically sophisticated manuscript that addresses a critical and timely challenge in computational systems biology. The integration of mean-field theory from statistical physics with attention mechanisms from deep learning is both novel and powerful. The paper's main strength lies in its principled approach to incorporating CCIs, moving beyond single-particle models to capture the collective behavior that is fundamental to biological processes. The results are compelling, and the demonstrated ability to uncover non-reciprocal interaction patterns is a significant advance with broad implications for studying complex biological systems. The work is of high quality and will undoubtedly be of great interest to the readership of PLOS Computational Biology. I recommend acceptance, contingent on the authors addressing the following points.

Response: We thank the reviewer for the insightful summary and positive assessment of our manuscript.

Major comments

R2.1: Scalability and Computational Complexity: The use of a self-attention mechanism has a computational complexity of $O(N^2)$ with respect to the number of cells (N). While the authors mention the model scales efficiently, this could become a bottleneck for very large datasets (e.g., $> 100,000$ cells per time point). The manuscript would be strengthened by a more detailed discussion of the model's computational performance, including runtime and memory usage as a function of cell number, and a comparison with the baseline models. Is it feasible to apply scIMF to atlas-scale datasets?

Response:

We thank the reviewer for raising this important point. While standard self-attention is $\mathcal{O}(N^2)$ in the number of cells N, scIMF adopts a mini-batch strategy and does not inherit this quadratic dependence and instead scales approximately linearly with N, since attention is confined to fixed-size batches and the only N–dependent term arises from the OT matching.

In the revised manuscript, we have added a detailed theoretical complexity analysis in the **Methods** section. We clarify that while standard self-attention is quadratic, our mini-batch implementation effectively linearizes the cost with respect to the total number of cells N. The revised text reads:

> "Since self-attention is $\mathcal{O}(N^2)$ in the number of cells N, scIMF adopts a mini-batch strategy to scale to large datasets by sampling B cells per iteration ($B \ll N$ in practice). In each iteration, the computational time complexity is dominated by two components: (i) the transformer-based drift term, which is $\mathcal{O}(B^2)$, and (ii) the computation of the Sinkhorn optimal transport loss between the predicted and observed cell distributions, which is $\mathcal{O}(BN)$. Consequently, for fixed batch size B (and a fixed number of Sinkhorn iterations), the overall complexity of scIMF grows approximately linearly with N, since attention is confined to fixed-size batches and the only N-dependent term arises from the entropically-regularized OT matching. The memory consumption likewise scales linearly with N, as evidenced by the approximately linear increase in time and memory usage with the total number of cells used in MEF dataset (Supplementary Text 1, Table S1 and Fig. S2)." (Lines 447–458)

To empirically validate this analysis, we measured the running time and memory usage on the MEF dataset. These results are now detailed in **Supplementary Text 1**, **Table S1**, and **Figure S2**.

Our empirical results confirm that the running time and memory consumption

increase linearly with the number of cells N. This demonstrates that scIMF scales efficiently and is well-suited for analyzing large-scale single-cell RNA-seq datasets.

R2.2: Interpretation of the Mean-Field Term: The drift term in the MV-SDE is elegantly split into an intra-cellular component (fintra) and an inter-cellular component (finter). However, the inter-cellular term is modeled entirely by the attention-based transformer encoder. Could the authors elaborate on whether this formulation has any potential limitations? For instance, does it assume all interactions are mediated through a single modality captured by the attention mechanism? A deeper discussion on the biological interpretation and potential constraints of this modeling choice would be beneficial.

Response:

We thank the reviewer for this insightful comment regarding the biological interpretation, limitations and constraints.

(1) Biological Interpretation and Modality. Biologically, we interpret the attention-based $\mathbf{f}_{\text{inter}}$ component as a dynamic proxy for the tissue microenvironment. By incorporating the empirical cell distribution, the model captures how collective signals exert a guiding force on individual differentiation paths—a feature inherently missing from single-particle formulations. We have clarified this in the **Overview of scIMF** subsection to explicitly link the mathematical choices to biological reality as follows:

> "··· These data are typically collected at multiple time points from distinct experimental individuals, resulting in population-level snapshots rather than matched cell trajectories. While previous Itô-SDE-based methods (e.g., PRESCIENT) effectively treat each cell as an independent particle, this approach assumes that a cell's fate is determined solely by its internal gene expression state. However, in multicellular systems, cell fate is strictly regulated by the extracellular environment and signals from neighboring cells. ··· Importantly, this mathematical structure mirrors biological reality: in developing tissues, a cell's trajectory is governed not only by its intracellular gene regulatory network (the intrinsic state) but also by the aggregate signaling milieu. By formally incorporating the empirical distribution into the drift term in Eq. 2, scIMF treats the "mean field" as a dynamic proxy for the tissue microenvironment. This enables the model to capture how collective signals exert a guiding

force on individual differentiation paths, a feature inherently missing from single-particle formulations." (Lines 60–76).

Regarding the reviewer's query on whether this assumes interactions are mediated by a single modality: while our primary implementation infers intercellular influences purely from gene expression data (a global component, $\mathbf{f}_{\text{global}}$), the architecture is designed to be extensible. As discussed in the revised manuscript, the interaction dynamics can be conceptually decomposed as $\mathbf{f}_{\text{inter}} = (1 - \alpha)\mathbf{f}_{\text{global}} + \alpha\mathbf{f}_{\text{L-R}}$. This allows for the integration of multi-modal priors, such as Ligand-Receptor databases, to mask or reweight the attention probabilities, ensuring that interactions align with known biological signaling pathways. We provided a detailed discussion on Page 12, Lines 300–313.

(2) Limitations and Constraints. We provide a detail discussion Limitations and Constraints in the revised manuscript on Pages 12–13, Lines 314–341.

First, the current formulation of scIMF adopts a homogeneous mean-field approximation, where the intercellular drift term $\mathbf{f}_{\text{inter}}$ is parameterized by a global attention encoder. Consequently, the same interaction kernel is implicitly applied across all cells and time points. While effective for standard scRNA-seq data, this design does not explicitly leverage modality-specific structures, such as spatial coordinates from spatially resolved transcriptomics or clonal relationships from lineage tracing. A key challenge moving forward is to integrate these complementary data modalities to impose structured constraints on the cell–cell relationship graph. Developing methods that incorporate such prior knowledge, while preserving the flexibility of attention-based architectures, remains an open and promising research direction for enhancing biological fidelity.

Second, our formulation employs a fixed, constant diffusion coefficient σ in Eq. (2), implying uniform noise levels across the population. Although this choice promotes numerical stability in Neural SDE solvers [30], it potentially oversimplifies the inter-cell/state-dependent variability inherent in transcriptomic dynamics. In future work, we plan to introduce a state-dependent diffusion term $\sigma(\mathbf{x}_t)$ to better capture heterogeneous stochasticity across distinct cell states, alongside the development of robust numerical methods to accommodate this increased complexity.

Third, the present scIMF framework relies on standard Optimal Transport (OT), which assumes the conservation of total probability mass over time. Consequently, the model does not explicitly account for cell proliferation or apoptosis, which may introduce bias when the underlying biological system undergoes significant expansion or contraction. A principled solution is to extend the framework to unbalanced OT formulations, such as the Wasserstein–Fisher–Rao distance employed in TIGON [31]. We view this as a natural evolution of scIMF and plan to explore this integration in future iterations.

> ***R2.3:*** Validation of Inferred Interactions: The paper compellingly shows that the inferred attention scores form asymmetric patterns in vivo and symmetric ones in vitro, which aligns with theoretical expectations. This is a powerful, albeit indirect, validation. To further solidify this key claim, could the authors attempt a more direct validation? For example, could the high-attention interactions be cross-referenced with known ligand-receptor pairs or signaling pathways that are expected to be active in those cell types at those developmental stages? While the model is designed to be prior-free, a post-hoc analysis connecting the learned interactions to known biology would significantly enhance the impact and credibility of the findings.

Response:

We thank the reviewer for this insightful comment. We agree that a direct validation connecting the learned attention scores to established biological priors is essential to enhance the credibility of our findings, even though scIMF is designed to be a prior-free method.

We have directly addressed this point through a detailed post-hoc analysis comparing scIMF's learned cell-type attention scores with interaction weights derived from the CellChat framework, which explicitly leverages a curated database of known zebrafish ligand-receptor (L-R) pairs and signaling pathways. This comparison, which serves as the direct validation requested, is presented in the main text (Pages 8,10, Lines 215–244) and Figure S1.

Summary of Validation and Interpretation

1. Quantitative Validation (Concordance): We quantified the agreement using the Spearman correlation coefficient (SCC) between the scIMF cell-type-by-cell-type attention matrix and the CellChat L-R interaction matrix for each developmental stage ($t = 3$ through $t = 11$). We observed moderate-to-strong positive concordance at key developmental stages, most prominently at $t = 6$ (SCC$= 0.55$) and $t = 10$ (SCC$= 0.41$). In these stages, the broadly increasing trend between the two scores demonstrates that scIMF successfully recovers the subset of interactions supported by established L-R evidence, thereby validating the biological relevance of the learned attention scores.

2. Interpreting Novelty (Divergence): The analysis also reveals valuable insights into scIMF's ability to identify new communication channels by examining stages with low correlation. The scatterplots exhibit two distinct modes of divergence (as detailed in the manuscript text):

- Vertical "Upward" Pattern (High scIMF / Low CellChat): This mode

(visible in $t = 3$–5) suggests that scIMF identifies putative interactions missed by the curated prior. This may occur when biologically active interactions are mediated by novel L-R pairs or non-canonical mechanisms that are not yet cataloged in the public database, which is a key advantage of a prior-free method.

- Shallow "Rightward" Pattern (Low scIMF / High CellChat): This mode (prominent in $t = 9$ and $t = 11$) suggests that scIMF can distinguish between potential communication (CellChat score based on L-R gene expression) and active signaling (the learned attention weight). This indicates that certain L-R pairs may be expressed in the relevant cell types but are not the primary drivers of cell-state change, which is a critical refinement in predicting functional communication.

In conclusion, this post-hoc analysis directly cross-references our learned attention scores with known biology (the CellChat L-R database), providing both quantitative validation and interpretable insights into novel communication channels that go beyond the limitations of prior-based methods.

Minor comments

> ***R2.4:*** Figure 2 Clarity: In Figure 2, the performance differences in the "Easy" tasks (interpolation) appear marginal between scIMF and some baselines (e.g., scNODE). The text accurately reflects that the advantage is more pronounced in harder, extrapolative tasks, but it may be worth explicitly noting in the figure caption that the benefits of modeling CCIs are most critical for future-state prediction.

Response: We thank the reviewer for the helpful suggestion. We now state explicitly in the Fig. 2 Caption on Page 6 that the benefits of modeling cell–cell interactions (CCIs) are most critical for future-state (extrapolative) prediction, while performance differences on "Easy" (interpolation) tasks are expected to be marginal.

> "Notably, for the ZB and MEF datasets, performance differences in the "Easy" (interpolation) tasks are relatively small across methods, whereas the advantages of explicitly modeling cell–cell interactions (CCIs) in scIMF become most pronounced in the "Medium" tasks, which require future-state (extrapolative) prediction."

> ***R2.5:*** Parameter Sensitivity: The manuscript does not include an analysis of the model's sensitivity to key hyperparameters (e.g., number of attention heads, embedding dimensions). A brief discussion or supplementary figure showing that the model's performance is robust across a reasonable range of hyperparameters would add to the paper's rigor.

Response:

We sincerely thank the reviewer for raising this crucial point regarding the parameter sensitivity. As recommended, we have now added evaluations of the model's sensitivity to two key hyperparameters: (i) the number of attention heads H, and (ii) the PCA embedding dimension r. Across all settings, the results demonstrate that scIMF exhibits robust performance with respect to these hyperparameter choices.

Accordingly, we have updated the **Materials and Methods** section of the revised manuscript to reflect these findings:

> "We set the number of attention heads to $H = 2$ for all three datasets analyzed in this study; notably, the performance of scIMF is robust to this choice (see Supplementary Text 2 and Table S2)." (Lines 459–461)

> "We set $r = 50$ for all three datasets analyzed in this study; notably, the performance of scIMF is robust to the choice of r (see Supplementary Text 2 and Fig. S3)." (Lines 469–471)

Detailed results of these analyses are provided in **Supplementary Text 2**, **Table S2**, and **Figure S3**.

> ***R2.6:*** Typographical Errors: Please check the manuscript for minor typographical errors. For example, in the held-out task description, "(4) Hard task" should likely be "(3) Hard task".

Response:

We thank the reviewer for their careful reading. We have corrected the numbering error in the held-out task description as suggested (changing "(4) Hard task" to "(3) Hard task") and have carefully proofread the entire manuscript to resolve other minor typographical errors.

Reviewer #3:

Overall comments

R3: This paper presents scIMF, a deep generative model for learning collective multicellular dynamics from temporal single-cell RNA-seq data. The method is conceptually grounded in the McKean–Vlasov stochastic differential equation framework, which provides a principled way to model population-level interactions. The incorporation of a cell-wise attention mechanism to capture nonlocal and asymmetric cell-cell interactions is an appealing idea and addresses a limitation of many existing approaches. The work is technically solid and demonstrates promising performance on several temporal scRNA-seq datasets, particularly in reconstructing gene expression at unobserved time points and inferring cellular velocities. The results suggest that scIMF can capture meaningful biological dependencies and provide interpretable insights into multicellular systems. Overall, this is a well-motivated and technically interesting study that contributes a novel perspective to modeling cell-cell interactions in dynamic single-cell data. The approach is promising but would benefit from more experimental justification and practical discussion of its computational and biological limitations. Here are the detailed comments.

Response: We appreciate the reviewer's thoughtful summary of our work and are pleased that they found our approach to modeling collective dynamics promising.

Major comments

R3.1: The authors emphasize temporal single-cell RNA sequencing, but it is unclear what exactly "temporal" refers to in this context. Currently, there are two main types of scRNA-seq data: (1) data collected from cells at a single time point from one individual, which require trajectory or pseudotime inference to reconstruct temporal information; and (2) data collected at multiple time points from different experimental individuals. If the authors refer to the first type, is scIMF sensitive to errors or fluctuations in pseudotime inference? If the second type is used, is scIMF sensitive to inter-individual variation among different experimental subjects? If another type of data is used, the authors should briefly clarify this in the manuscript.

Response:

We thank the reviewer for this opportunity to clarify the data types used in our study and the associated modeling assumptions.

To clarify, in this work, "temporal scRNA-seq" refers specifically to datasets collected at multiple observed time points from different experimental individuals (Type 2). We have explicitly clarified this definition in the **Overview of scIMF** subsection of the revised manuscript:

> "These data are typically collected at multiple time points from distinct experimental individuals, resulting in population-level snapshots rather than matched cell trajectories." (Lines 60–62).

Regarding the reviewer's query on sensitivity to inter-individual variation (which is inherent to Type 2 data), our current framework addresses stochasticity via the diffusion term in the MV-SDE. However, scIMF currently employs a fixed constant diffusion coefficient σ. This implies that the model treats noise—including inter-individual variability—uniformly across the population, rather than explicitly modeling individual-specific batch effects or state-dependent variances. We have expanded on this limitation and our plans to address it via state-dependent diffusion in the **Discussion** section:

> "Second, our formulation employs a fixed, constant diffusion coefficient σ in Eq. 2, implying uniform noise levels across the population. Although this choice promotes numerical stability in Neural SDE solvers [30], it potentially oversimplifies the inter-cell/state-dependent variability inherent in transcriptomic dynamics. In future work, we plan to introduce a state-dependent diffusion term $\sigma(\mathbf{x}_t)$ to better capture heterogeneous stochasticity across distinct cell states, alongside the development of robust numerical methods to accommodate this increased complexity." (Lines 327–333).

R3.2: Since cellular dynamics evolve continuously over time while the observed data are discrete, are there potential latent states that are unobserved or unmodeled? Would this omission introduce bias when computing optimal transport distances?

Response:

We thank the reviewer for this insightful question regarding the discrepancy between continuous biological evolution and discrete observation.

Indeed, there may exist latent cellular states that are unobserved or not explicitly modeled in the current framework. Specifically, because cell populations change dynamically over time due to division and apoptosis, a growth term capturing these net population changes is often necessary to fully model the underlying biological dynamics. However, the current scIMF framework relies on standard Optimal Transport (OT), which enforces the conservation of total probability mass over time. Consequently, scIMF does not explicitly account for cell proliferation or loss during development.

We acknowledge that this omission may introduce bias when the true dynamics involve substantial population expansion or contraction between observed time points. A principled solution to address this limitation is to extend the framework to unbalanced OT formulations, such as the Wasserstein–Fisher–Rao distance introduced in methods like TIGON (Sha et al., 2024). We view this as a natural and important extension of scIMF and plan to explore it in future work.

In the revised manuscript, we have explicitly stated this assumption and added a discussion of this limitation in the **Discussion** section:

> "Third, the present scIMF framework relies on standard optimal transport (OT), which assumes the conservation of total probability mass over time. Consequently, the model does not explicitly account for cell proliferation or apoptosis, which may introduce bias when the underlying biological system undergoes significant expansion or contraction. A principled solution is to extend the framework to unbalanced OT formulations, such as the Wasserstein–Fisher–Rao distance employed in TIGON [31]. We view this as a natural evolution of scIMF and plan to explore this integration in future iterations." (Lines 334–341).

R3.3: The algorithm employs modeling frameworks such as the McKean–Vlasov stochastic differential equation and mean-field games. Although the empirical results support their effectiveness, it would be helpful if the authors could provide biological intuition to explain why these methods yield better results in this context.

Response:

We thank the reviewer for this insightful comment. We agree that connecting the mathematical formalism to biological intuition is crucial for interpreting

the model's success. The superior performance of the McKean–Vlasov SDE (MV-SDE) and mean-field game frameworks stems from their ability to model cells not as isolated entities, but as members of an interacting community—a fundamental feature of multicellular biology.

In the revised manuscript, we have clarified this intuition in the **Overview of scIMF** subsection to explicitly link the mathematical choices to biological reality. The core biological justifications are as follows:

> "⋯ While previous Itô-SDE-based methods (e.g., PRESCIENT) effectively treat each cell as an independent particle, this approach assumes that a cell's fate is determined solely by its internal gene expression state. However, in multicellular systems, cell fate is strictly regulated by the extracellular environment and signals from neighboring cells. ⋯ Importantly, this mathematical structure mirrors biological reality: in developing tissues, a cell's trajectory is governed not only by its intracellular gene regulatory network (the intrinsic state) but also by the aggregate signaling milieu. By formally incorporating the empirical distribution into the drift term in Eq. 2, scIMF treats the "mean field" as a dynamic proxy for the tissue microenvironment. This enables the model to capture how collective signals exert a guiding force on individual differentiation paths, a feature inherently missing from single-particle formulations." (Lines 62–76).

R3.4: The authors mention dimensionality reduction in preprocessing, but the choice of method and dimensionality is not further discussed. How sensitive are the results to these choices? How should one select the dimensionality in practice?

Response:

We thank the reviewer for raising this important point regarding the choice of dimensionality reduction and its impact on modeling.

In all experiments, we utilized Principal Component Analysis (PCA) as the sole dimensionality reduction step, performing modeling in an r-dimensional latent space. We selected PCA specifically for its linearity and its tendency to preserve the global structure of the data. This preservation is critical for the reliability of the Wasserstein distances used in our evaluation. By contrast, non-linear embeddings (such as t-SNE or UMAP) often distort pairwise distances and global geometry, thereby complicating OT-based comparisons.

Regarding the selection of dimensionality, we followed standard practice by setting $r = 50$ for all datasets. This choice provides a compact representation while retaining the majority of the variance among highly variable genes.

To address the reviewer's query regarding sensitivity, we have added evaluations of the model's performance across different PCA embedding dimensions r. Our results demonstrate that scIMF exhibits robust performance with respect to r across a wide range of values.

Accordingly, we have updated the **Data Preprocessing** subsection within **Materials and Methods** in the revised manuscript to reflect these findings:

> "We set $r = 50$ for all three datasets analyzed in this study; notably, the performance of scIMF is robust to the choice of r (see Supplementary Text 2 and Fig. S3)." (Lines 469–471)

Detailed results of these analyses are provided in **Supplementary Text 2** and **Figure S3**.

> ***R3.5:*** Some symbols in the equations are not clearly defined or explained—for instance, $\rho(x,t)$. Also, in Equation (2), $x_{t_0} = \rho_{t_0}$, the left-hand side is a numerical value, while the right-hand side is a distribution. Even if it represents a degenerate (single-point) distribution, the notation seems nonstandard and should be clarified.

Response:

We thank the reviewer for their careful attention to the mathematical notation and for pointing out these issues.

First, we apologize for the typographical error in the initial condition statement of Equation (2). The correct expression is indeed:

$$\mathbf{x}_{t_0} \sim \rho_{t_0},$$

which indicates that the random variable $\mathbf{x}_{t_0}$ is drawn from the distribution ρ_{t_0}. We have corrected this notation throughout the manuscript. We confirm that this revision is purely notational and does not affect any derivations, algorithms, or results.

Second, we have explicitly defined all symbols appearing in the MV-SDE formulation to ensure clarity. In the revised **Materials and Methods** section, we added the following definition for ρ_t:

> "Here, ρ_t (or equivalently $\rho(\mathbf{x}, t)$) denotes the population-level probability distribution of cellular state $\mathbf{x}$ at time t." (Lines 350–351)

> ***R3.6:*** From Fig.2, the results on the first two in vivo datasets appear better than those on the in vitro dataset. Is there any biological or methodological explanation for this difference?

Response:

We thank the reviewer for this insightful question regarding the performance differences between datasets.

We believe the observed difference primarily reflects the underlying biological context rather than a methodological artifact. In in vivo developmental systems (such as the zebrafish and MEF datasets), cells reside within a rich and spatially structured microenvironment containing diverse neighbors and lineage-specific signaling sources. These factors generate strong, non-local, and often asymmetric cell–cell interactions (CCIs) that drive the system far from equilibrium. Because scIMF explicitly models these population-level influences via an attention-based mean-field term, it derives significant benefit in these complex settings.

By contrast, the in vitro culture system (Panc dataset) is substantially more homogeneous. In this context, cell signaling is often diluted by well-mixed media and niche complexity is markedly lower, resulting in interaction signals that are weaker and closer to reciprocal or quasi-equilibrium dynamics. In such settings, the additional expressiveness of scIMF provides a smaller relative advantage over baselines (like PI-SDE) that do not explicitly model CCIs, as the system behavior is less dependent on complex intercellular dependencies.

In the revised manuscript, we have emphasized this interpretation in the **Results** section:

> "Taken together, the underlying dynamics of in vitro β-cell differentiation appear to approach equilibrium or quasi-equilibrium, driven by reciprocal cell-cell interactions [20,21]. In the homogeneous in vitro environment (Panc dataset), where cell signaling is diluted and niche complexity is low. In such settings, the capacity of scIMF to model CCIs yields a smaller marginal gain over baselines like PI-SDE, as evidenced by their comparable performance in Fig. 2E–F.

Conversely, in vivo systems (e.g., zebrafish and MEF datasets) feature complex microenvironments with strong, asymmetric CCIs that drive the system far from equilibrium. Because scIMF captures these population-level influences via an attention-based mean-field term, it demonstrates superior performance in these highly dynamic settings (Fig. 2A–D)." (Lines 268–273)

R3.7: The brief description of the empirical datasets is placed on page 11. It might be better to move this section earlier, as some parts of the manuscript (for example, the discussion of "held-out time points" on page 4) are difficult to follow without this context.

Response:

We thank the reviewer for this constructive suggestion to improve the manuscript's organization.

We agree that introducing the datasets earlier is essential for understanding the experimental setup, particularly the held-out evaluation tasks. Accordingly, in the revised manuscript, we have moved the detailed descriptions of all three datasets from the **Data Preprocessing** subsection (in **Materials and Methods**) to the beginning of the **Results** section.

This reorganization ensures that readers have the necessary context regarding the biological systems and sampling intervals before encountering the discussion of held-out time points. We believe this change significantly enhances the logical flow and readability of the paper.
